# Supplementary material for: Serially assessed bisphenol A and phthalate exposure and association with kidney function in children with chronic kidney disease in the US and Canada: A longitudinal cohort study
Source: PLoS Med. 2020 Oct 14;17(10):e1003384. doi: 10.1371/journal.pmed.1003384 (PMC7556524; doi:10.1371/journal.pmed.1003384)
Supplement: S1 Fig — (DOCX) [file pmed.1003384.s002.docx]

**S1 Fig**. Modeling strategy for linear mixed-effects models with random intercept and AR(1) error term

x: exposure y: outcome z: confounders

Fit $Y\sim\beta_{1}x+\beta_{2}t+\beta_{3}t^{2}+\beta_{4}z$

No $\beta_{1} is significant$? Yes

Report $\beta_{1}$ in table

Fit $Y\sim\beta_{1}x+\beta_{2}t+\beta_{3}t^{2}+\beta_{4}z+\beta_{5}x\cdot t+\beta_{6}x\cdot t^{2}$

Yes $\beta_{5} and \beta_{6}$ are significant? No

Report $\beta_{1}$ in table

Report $\beta_{1}$ in table (representing effect at t=0)

Plot time-specific effect in Figure
