# Supplementary material for: Serially assessed bisphenol A and phthalate exposure and association with kidney function in children with chronic kidney disease in the US and Canada: A longitudinal cohort study
Source: PLoS Med. 2020 Oct 14;17(10):e1003384. doi: 10.1371/journal.pmed.1003384 (PMC7556524; doi:10.1371/journal.pmed.1003384)
Supplement: S1 Table — (DOCX) [file pmed.1003384.s003.docx]

| **S1 Table**. Intraclass correlation coefficients (ICC) and 95% Confidence Intervals (CI) for ln-transformed chemical exposures and outcomes over time | |
| --- | --- |
| ***Exposures*** | **ICC [95% CI]** |
| BPA | 0.13 [0.09, 0.17] |
| PA | 0.15 [0.12, 0.20] |
| LMW | 0.39 [0.34, 0.43] |
| HMW | 0.26 [0.22, 0.31] |
| DEHP | 0.22 [0.18, 0.27] |
| DOP | 0.12 [0.08, 0.16] |
| ***Outcomes*** |  |
| eGFR | 0.83 [0.81, 0.85] |
| Ln(UPCR) | 0.77 [0.74, 0.79] |
| SBP Z-score | 0.51 [0.47, 0.55] |
| DBP Z-score | 0.42 [0.38, 0.47] |
| Ln(8-OHdG) | 0.32 [0.27, 0.36] |
| Ln(F_2_-isoprostane)^a^ | <0.01 |
| Ln(NGAL) | 0.60 [0.56, 0.64] |
| Ln(KIM-1) | 0.50 [0.46, 0.54] |
| Abbreviations: BPA: bisphenol A; PA: phthalic acid; LMW: low-molecular weight phthalates; HMW: high molecular weight phthalates; DEHP: di (2-ethylhexyl) phthalate; DOP: dioctyl phthalate; eGFR: estimated glomerular filtration rate; UPCR: urinary protein to creatinine ratio; IQR: interquartile range; SBP: systolic blood pressure; DBP: diastolic blood pressure; 8-OHdG: 8-hydroxy-2’-deoxyguanosine; NGAL: Neutrophil gelatinase-associated lipocalin; KIM-1: Kidney Injury Molecule-1  ^a^N=1287 samples had data on F_2_-isoprostane and the median (IQR) number of visits for subjects with available data=2 (1,3), thus limiting ICC calculations. | |
