# Supplementary material for: Serially assessed bisphenol A and phthalate exposure and association with kidney function in children with chronic kidney disease in the US and Canada: A longitudinal cohort study
Source: PLoS Med. 2020 Oct 14;17(10):e1003384. doi: 10.1371/journal.pmed.1003384 (PMC7556524; doi:10.1371/journal.pmed.1003384)
Supplement: S2 Table — (DOCX) [file pmed.1003384.s004.docx]

| **S2 Table**. Associations between calendar year and urinary chemical concentrations over time from linear mixed-effects models | | | | |
| --- | --- | --- | --- | --- |
| Exposure | Parameter | Estimate | 95% CI | p-value |
| BPA | $\beta_{2}$ | -0.042 | -0.062, -0.022 | <0.001 |
|  | $\beta_{3}$ | 0.005 | -0.020, 0.030 | 0.707 |
| PA | $\beta_{2}$ | -0.009 | -0.029, 0.011 | 0.352 |
|  | $\beta_{3}$ | 0.195 | 0.171, 0.219 | <0.001 |
| LMW | $\beta_{2}$ | -0.040 | -0.064, -0.016 | 0.001 |
|  | $\beta_{3}$ | -0.006 | -0.028, 0.016 | 0.561 |
| HMW | $\beta_{2}$ | -0.052 | -0.074, -0.030 | <0.001 |
|  | $\beta_{3}$ | 0.054 | 0.030, 0.078 | <0.001 |
| DEHP | $\beta_{2}$ | -0.066 | -0.086, -0.046 | <0.001 |
|  | $\beta_{3}$ | 0.045 | 0.021, 0.069 | <0.001 |
| DOP | $\beta_{2}$ | -0.089 | -0.11, -0.069 | <0.001 |
|  | $\beta_{3}$ | -0.193 | -0.22, -0.169 | <0.001 |
| Estimates were derived from the following model: $Y_{ij}= \beta_{1}+a_{i}+\beta_{2}x_{i1}+ \beta_{3}\left( x_{ij}-x_{i1} \right)+\varepsilon_{ij}$ where $Y_{ij}$ is the ln-transformed urinary concentration of each exposure, $x_{ij}$ is the year of $j$-th observation for subject $i$, $x_{i1}$ is the year of 1^st^ observation for subject $i$, and $a_{i}$ is a random intercept. Hence, ($x_{ij}-x_{i1}$) represents change in years from the first observation to $j$th observation. $\beta_{2}$ represents the effect of year of enrollment on the average change in $Y_{ij}$. $\beta_{3}$ represents the longitudinal effect of year in the expected change in $Y_{ij}$ for a given subject. | | | | |
