# Supplementary material for: Serially assessed bisphenol A and phthalate exposure and association with kidney function in children with chronic kidney disease in the US and Canada: A longitudinal cohort study
Source: PLoS Med. 2020 Oct 14;17(10):e1003384. doi: 10.1371/journal.pmed.1003384 (PMC7556524; doi:10.1371/journal.pmed.1003384)
Supplement: S3 Table — (DOCX) [file pmed.1003384.s005.docx]

| **S3 Table**. Time-specific estimates for the associations between ln-transformed chemical exposures and ln-transformed tubular injury biomarkers from linear mixed-effects models as shown in Figure 1 | | | | | | |
| --- | --- | --- | --- | --- | --- | --- |
|  | PA and KIM-1 |  | HMW and KIM-1 |  | DOP and KIM-1 |  |
|  | Estimate (95% CI) | p | Estimate (95% CI) | p | Estimate (95% CI) | p |
| Baseline | -0.006 (-0.083, 0.070) | 0.8723 | 0.126 (0.031, 0.220) | 0.0093 | 0.303 (0.206, 0.400) | <0.0001 |
| Visit 1 | 0.150 (0.082, 0.217) | <0.0001 | 0.211 (0.141, 0.282) | <0.0001 | 0.287 (0.215, 0.358) | <0.0001 |
| Visit 2 | 0.341 (0.245, 0.436) | <0.0001 | 0.295 (0.213, 0.377) | <0.0001 | 0.246 (0.161, 0.331) | <0.0001 |
| Visit 3 | 0.370 (0.268, 0.472) | <0.0001 | 0.261 (0.172, 0.351) | <0.0001 | 0.194 (0.097, 0.291) | <0.0001 |
| Visit 4 | 0.238 (0.114, 0.361) | 0.0002 | 0.110 (0.013, 0.208) | 0.0259 | 0.131 (0.031, 0.232) | 0.0106 |
| Visit 5 | -0.056 (-0.294, 0.181) | 0.6421 | -0.158 (-0.334, 0.018) | 0.0787 | 0.057 (-0.106, 0.219) | 0.493 |

Estimates correspond to a 1-standard deviation change in each ln-transformed chemical exposure.
