# Supplementary material for: Serially assessed bisphenol A and phthalate exposure and association with kidney function in children with chronic kidney disease in the US and Canada: A longitudinal cohort study
Source: PLoS Med. 2020 Oct 14;17(10):e1003384. doi: 10.1371/journal.pmed.1003384 (PMC7556524; doi:10.1371/journal.pmed.1003384)
Supplement: S4 Table — (DOCX) [file pmed.1003384.s006.docx]

| **S4 Table**. Time-specific estimates for the associations between ln-transformed chemical exposures and ln-transformed oxidative stress biomarkers from linear mixed-effects models as shown in Figure 2 | | | | |
| --- | --- | --- | --- | --- |
|  | BPA and 8-OHdG |  | PA and 8-OHdG |  |
|  | Estimate (95% CI) | p | Estimate (95% CI) | p |
| Baseline | 0.050 (-0.002, 0.102) | 0.059 | 0.097 (0.054, 0.140) | <0.0001 |
| Visit 1 | 0.072 (0.032, 0.112) | 0.0004 | 0.152 (0.115, 0.190) | <0.0001 |
| Visit 2 | 0.109 (0.062, 0.156) | <0.0001 | 0.234 (0.181, 0.288) | <0.0001 |
| Visit 3 | 0.140 (0.088, 0.191) | <0.0001 | 0.279 (0.222, 0.337) | <0.0001 |
| Visit 4 | 0.163 (0.117, 0.209) | <0.0001 | 0.287 (0.224, 0.351) | <0.0001 |
| Visit 5 | 0.179 (0.110, 0.248) | <0.0001 | 0.258 (0.145, 0.371) | <0.0001 |
|  |  |  |  |  |
|  | BPA and F_2_-isoprostane |  | DEHP and F_2_-isoprostane |  |
|  | Estimate (95% CI) | p | Estimate (95% CI) | p |
| Baseline | 0.111 (-0.146, 0.367) | 0.398 | -0.103 (-0.351, 0.144) | 0.4141 |
| Visit 1 | 0.091 (-0.097, 0.280) | 0.341 | -0.010 (-0.195, 0.176) | 0.9196 |
| Visit 2 | 0.099 (-0.044, 0.242) | 0.1736 | 0.142 (-0.018, 0.303) | 0.0817 |
| Visit 3 | 0.168 (0.027, 0.309) | 0.0195 | 0.248 (0.088, 0.408) | 0.0024 |
| Visit 4 | 0.298 (0.176, 0.421) | <0.0001 | 0.306 (0.160, 0.452) | <0.0001 |
| Visit 5 | 0.490 (0.321, 0.659) | <0.0001 | 0.317 (0.082, 0.552) | 0.0082 |

Estimates correspond to a 1-standard deviation change in each ln-transformed chemical exposure.
