# Supplementary material for: Serially assessed bisphenol A and phthalate exposure and association with kidney function in children with chronic kidney disease in the US and Canada: A longitudinal cohort study
Source: PLoS Med. 2020 Oct 14;17(10):e1003384. doi: 10.1371/journal.pmed.1003384 (PMC7556524; doi:10.1371/journal.pmed.1003384)
Supplement: S5 Table — (DOCX) [file pmed.1003384.s007.docx]

| **S5 Table**. Associations between cumulative average ln-transformed chemical exposures and eGFR, ln-transformed urinary protein-to-creatinine ratio, SBP z-score, and DBP z-score from linear mixed-effects models | | | | | | | | | | | | |
| --- | --- | --- | --- | --- | --- | --- | --- | --- | --- | --- | --- | --- |
|  | eGFR | | | SBP Z-score | | | DBP Z-score | | | Ln-UPCR | | |
|  | β | 95% CI | p | β | 95% CI | p | β | 95% CI | p | β | 95% CI | p |
| BPA | -0.547 | -1.222, 0.128 | 0.112 | 0.012 | -0.045, 0.069 | 0.682 | 0.012 | -0.041, 0.065 | 0.644 | 0.015 | -0.044, 0.074 | 0.621 |
| PA | -0.308 | -0.916, 0.300 | 0.321 | 0.001 | -0.048, 0.050 | 0.971 | 0.007 | -0.038, 0.052 | 0.763 | -0.007 | -0.060, 0.046 | 0.781 |
| LMW | 1.562 | 0.658, 2.466 | 0.001 | -0.005 | -0.078, 0.068 | 0.902 | -0.013 | -0.078, 0.052 | 0.692 | -0.050 | -0.126, 0.026 | 0.206 |
| HMW | 0.813 | -0.195, 1.821 | 0.114 | 0.022 | -0.060, 0.104 | 0.596 | 0.003 | -0.072, 0.078 | 0.927 | -0.039 | -0.125, 0.047 | 0.368 |
| DEHP | 0.501 | -0.360, 1.362 | 0.254 | 0.03 | -0.041, 0.101 | 0.406 | 0.009 | -0.056, 0.074 | 0.783 | -0.011 | -0.086, 0.064 | 0.765 |
| DOP | -0.432 | -1.281, 0.417 | 0.318 | -0.008 | -0.079, 0.063 | 0.817 | -0.006 | -0.071, 0.059 | 0.857 | -0.041 | -0.114, 0.032 | 0.276 |

Estimates correspond to a log-unit in each ln-transformed chemical exposure.
