# Supplementary material for: Serially assessed bisphenol A and phthalate exposure and association with kidney function in children with chronic kidney disease in the US and Canada: A longitudinal cohort study
Source: PLoS Med. 2020 Oct 14;17(10):e1003384. doi: 10.1371/journal.pmed.1003384 (PMC7556524; doi:10.1371/journal.pmed.1003384)
Supplement: S6 Table — (DOCX) [file pmed.1003384.s008.docx]

| **S6 Table**. Associations between cumulative average ln-transformed chemical exposures and ln-transformed kidney injury biomarkers from linear mixed-effects models | | | | | | | |
| --- | --- | --- | --- | --- | --- | --- | --- |
|  | NGAL | | |  | KIM-1 | | |
|  | β | 95% CI | p |  | β | 95% CI | p |
| BPA | 0.075 | -0.035, 0.185 | 0.176 | BPA | 0.093 | 0.013, 0.173 | 0.022 |
| PA | 0.169 | 0.073, 0.265 | 0.001 | PA^*^ | 0.035 | -0.038, 0.108 | 0.342 |
| LMW | -0.039 | -0.180, 0.102 | 0.587 | LMW | 0.176 | 0.074, 0.278 | 0.001 |
| HMW | 0.183 | 0.026, 0.340 | 0.022 | HMW^*^ | 0.159 | 0.030, 0.288 | 0.016 |
| DEHP | 0.140 | 0.005, 0.275 | 0.043 | DEHP | 0.131 | 0.033, 0.229 | 0.009 |
| DOP | 0.091 | -0.044, 0.226 | 0.185 | DOP | 0.331 | 0.233, 0.429 | <0.0001 |
| ^*^Exposure has significant interaction (p<0.05) with time and estimate for exposure at baseline is presented; time-specific estimates shown in Table S6.  Estimates correspond to a log-unit in each ln-transformed chemical exposure. | | | | | | | |
