# Supplementary material for: Serially assessed bisphenol A and phthalate exposure and association with kidney function in children with chronic kidney disease in the US and Canada: A longitudinal cohort study
Source: PLoS Med. 2020 Oct 14;17(10):e1003384. doi: 10.1371/journal.pmed.1003384 (PMC7556524; doi:10.1371/journal.pmed.1003384)
Supplement: S7 Table — (DOCX) [file pmed.1003384.s009.docx]

| **S7 Table**. Time-specific estimates for associations between cumulative average ln-transformed chemical exposures and ln-transformed biomarkers of tubular injury from linear mixed-effects models | | | | |
| --- | --- | --- | --- | --- |
|  | PA and KIM-1 |  | HMW and KIM-1 |  |
|  | Estimate (95% CI) | p | Estimate (95% CI) | p |
| Baseline | 0.035 (-0.037, 0.106) | 0.3421 | 0.159 (0.030, 0.287) | 0.0154 |
| Visit 1 | 0.151 (0.073, 0.228) | 0.0001 | 0.269 (0.151, 0.387) | <0.0001 |
| Visit 2 | 0.293 (0.175, 0.410) | <0.0001 | 0.378 (0.216, 0.539) | <0.0001 |
| Visit 3 | 0.315 (0.178, 0.452) | <0.0001 | 0.336 (0.153, 0.520) | 0.0003 |
| Visit 4 | 0.218 (0.043, 0.393) | 0.0145 | 0.146 (-0.072, 0.364) | 0.1886 |
| Visit 5 | 0.002 (-0.291, 0.294) | 0.9907 | -0.194 (-0.551, 0.164) | 0.2881 |

Estimates correspond to a log-unit in each ln-transformed chemical exposure.
