# Supplementary material for: Serially assessed bisphenol A and phthalate exposure and association with kidney function in children with chronic kidney disease in the US and Canada: A longitudinal cohort study
Source: PLoS Med. 2020 Oct 14;17(10):e1003384. doi: 10.1371/journal.pmed.1003384 (PMC7556524; doi:10.1371/journal.pmed.1003384)
Supplement: S8 Table — (DOCX) [file pmed.1003384.s010.docx]

| **S8 Table**. Associations between cumulative average ln-transformed chemical exposures and ln-transformed oxidative stress biomarkers from linear mixed-effects models | | | | | | | |
| --- | --- | --- | --- | --- | --- | --- | --- |
|  | Ln-8-OHdG | |  |  | Ln-F_2_-isoprostane | |  |
|  | β | 95% CI | p |  | β | 95% CI | p |
| BPA | 0.085 | 0.044, 0.126 | <0.0001 | BPA^*^ | 0.106 | -0.109, 0.321 | 0.336 |
| PA^*^ | 0.119 | 0.081, 0.157 | <0.0001 | PA^*^ | -0.218 | -0.536, 0.100 | 0.179 |
| LMW | 0.219 | 0.168, 0.270 | <0.0001 | LMW | 0.211 | 0.076, 0.346 | 0.002 |
| HMW^*^ | 0.255 | 0.186, 0.324 | <0.0001 | HMW | -0.09 | -0.259, 0.079 | 0.292 |
| DEHP | 0.184 | 0.133, 0.235 | <0.0001 | DEHP | -0.064 | -0.215, 0.087 | 0.401 |
| DOP^*^ | 0.063 | 0.003, 0.124 | 0.039 | DOP^*^ | -0.116 | -0.389, 0.157 | 0.406 |
| ^*^Exposure has significant interaction (p<0.05) with time and estimate for exposure at baseline is presented; time-specific estimates shown in Table S8.  Estimates correspond to a log-unit in each ln-transformed chemical exposure. | | | | | | | |
