# Supplementary material for: Serially assessed bisphenol A and phthalate exposure and association with kidney function in children with chronic kidney disease in the US and Canada: A longitudinal cohort study
Source: PLoS Med. 2020 Oct 14;17(10):e1003384. doi: 10.1371/journal.pmed.1003384 (PMC7556524; doi:10.1371/journal.pmed.1003384)
Supplement: S9 Table — (DOCX) [file pmed.1003384.s011.docx]

| **S9 Table.** Time-specific estimates for associations between cumulative average ln-transformed chemical exposures and ln-transformed oxidative stress biomarkers from linear mixed-effects models | | | | | | |
| --- | --- | --- | --- | --- | --- | --- |
|  | PA and 8-OHdG | | HMW and 8-OHdG | | DOP and 8-OHdG | |
|  | Estimate (95% CI) | p | Estimate (95% CI) | p | Estimate (95% CI) | p |
| Baseline | 0.119 (0.081, 0.157) | <0.0001 | 0.255 (0.186, 0.324) | <0.0001 | 0.063 (0.003, 0.124) | 0.0393 |
| Visit 1 | 0.143 (0.104, 0.183) | <0.0001 | 0.209 (0.149, 0.270) | <0.0001 | 0.032 (-0.022, 0.085) | 0.2444 |
| Visit 2 | 0.186 (0.126, 0.247) | <0.0001 | 0.163 (0.080, 0.247) | 0.0001 | 0.006 (-0.068, 0.080) | 0.8669 |
| Visit 3 | 0.220 (0.150, 0.291) | <0.0001 | 0.177 (0.081, 0.273) | 0.0003 | 0.031 (-0.054, 0.116) | 0.472 |
| Visit 4 | 0.246 (0.160, 0.332) | <0.0001 | 0.251 (0.144, 0.359) | <0.0001 | 0.106 (0.011, 0.201) | 0.029 |
| Visit 5 | 0.263 (0.120, 0.405) | 0.0003 | 0.385 (0.217, 0.553) | <0.0001 | 0.231 (0.081, 0.381) | 0.0025 |
|  |  | |  | |  | |
|  | BPA and F_2_-isoprostane | | PA and F_2_-isoprostane | | DOP and F_2_-isoprostane | |
|  | Estimate (95% CI) | p | Estimate (95% CI) | p | Estimate (95% CI) | p |
| Baseline | 0.106 (-0.109, 0.321) | 0.3357 | -0.218 (-0.536, 0.100) | 0.179 | -0.116 (-0.389, 0.157) | 0.406 |
| Visit 1 | 0.028 (-0.136, 0.193) | 0.7354 | -0.188 (-0.416, 0.040) | 0.1054 | -0.052 (-0.259, 0.154) | 0.6203 |
| Visit 2 | -0.026 (-0.197, 0.145) | 0.7646 | -0.097 (-0.274, 0.079) | 0.2808 | 0.079 (-0.124, 0.282) | 0.4455 |
| Visit 3 | 0.053 (-0.128, 0.234) | 0.5662 | 0.035 (-0.144, 0.215) | 0.7002 | 0.216 (0.004, 0.428) | 0.0461 |
| Visit 4 | 0.265 (0.095, 0.435) | 0.0022 | 0.209 (0.022, 0.397) | 0.0289 | 0.358 (0.150, 0.567) | 0.0007 |
| Visit 5 | 0.611 (0.341, 0.882) | <0.0001 | 0.424 (0.091, 0.758) | 0.0127 | 0.506 (0.160, 0.853) | 0.0042 |

Estimates correspond to a log unit change in each ln-transformed chemical exposure.
