# Supplementary material for: Serially assessed bisphenol A and phthalate exposure and association with kidney function in children with chronic kidney disease in the US and Canada: A longitudinal cohort study
Source: PLoS Med. 2020 Oct 14;17(10):e1003384. doi: 10.1371/journal.pmed.1003384 (PMC7556524; doi:10.1371/journal.pmed.1003384)
Supplement: S1 Text — (DOCX) [file pmed.1003384.s012.docx]

**S1 Text.** Analysis plan

**Serially assessed bisphenol A or phthalate exposure and association with kidney function in children with chronic kidney disease in the US and Canada: a longitudinal cohort study**

**Specific Aims**

Aim 1: To determine if BPA and phthalate exposures are associated with accelerated decline in renal function in children with CKD.

H1. Urinary BPA and DEHP metabolites at study entry will be associated with a higher rate of decline in eGFR among children with CKD.

Aim 2: To determine if BPA and phthalate exposures are associated with the level of proteinuria, BP, and excretion of biomarkers of tubular injury in children with CKD.

H2a. Change in proteinuria is associated with urinary BPA and DEHP at study entry.

H2b. Urinary BPA and DEHP metabolites at study entry will be associated with persistent prehypertension or hypertension at the last study visit, controlled for entry BP.

H2c. Urinary BPA and DEHP metabolites at study entry will be associated with increases in urinary excretion of kidney injury molecule-1 and neutrophil gelatinase associated lipocalcin.

Aim 3: To determine if BPA and phthalate exposures are associated with increases in urinary markers of oxidative stress, consistent with the proposed biological mechanism of disease.

H3. Urinary BPA and DEHP metabolites are associated with increases in urinary markers of oxidative stress.

**Methods**

Analysis of phthalate metabolites

We will assess biomarkers in groups corresponding to their use in product categories. We will express the LMW concentration as the sum of molar concentrations of mono-ethyl phthalate (MEP), mono-n-butylphthalate (MBP), and mono-isobutyl phthalate (MiBP), expressed on the basis of urinary creatinine to correct for urinary dilution (i.e. moles LMW/g creatinine). The HMW concentration will be calculated as the sum of molarities of mono-(2-ethyl-5-carboxypentyl) phthalate (MECPP), mono-(3-carboxypropyl) phthalate (MCPP), mono-(2-ethyl-5-hydroxyhexyl) phthalate (MEHHP), mono-(2-ethyl-5-oxohexyl) phthalate (MEOHP), mono-(2-ethylhexyl) phthalate (MEHP) and monobenzylphthalate (MBzP), divided by the creatinine concentration of the sample. Finally, we will calculate the DEHP metabolite concentration by adding molarities of MEHP, MECPP, MEHHP and MEOHP, and dividing by the creatinine concentration of the sample.

Aim 1

eGFR will be determined using the modified Schwartz formula. Multivariable linear models will examine eGFR as the primary dependent variable. We will use mixed-effect models for repeatedly measured continuous outcomes using random effects to account for individual level heterogeneity. Initial models will examine concentrations of BPA and phthalate metabolite groups separately, and final models will examine multiple (BPA and phthalate) exposures together based upon their significance at p<0.10.

We appreciate that glomerular and non-glomerular CKD patients generally experience different patterns in eGFR loss. The best data on these differential declines are from the CKiD study, which document approximately 1-2 ml/min/year in non-glomerular disease and 3-5 ml/min/yr in glomerular CKD. Other potential confounders include BMI category (which has been associated with BPA and phthalate exposures, and decline in eGFR in CKD). Studies have been unable to test the hypothesis that regional differences in processed versus fresh foods might predict both BPA/phthalate exposure and progression of CKD. However, racial/ethnic differences in phthalate and BPA exposures have been identified, just as they have for CKD. We therefore propose to examine potential confounding by race/ethnicity. Given sex differences in some phthalate exposures, we will control for sex as well, and examine sex as a possible predilection to BPA/phthalate-associated effects.

Treatment covariates that will be available for all study participants will include use of agents that inhibit the renin-angiotensin axis and immunosuppressive agents and surgical procedures in patients with non-glomerular CKD. Other covariates available in CKiD that may

improve model fit, even though we hypothesize them not to be potential confounders are: prematurity and birth weight. We will also control for age/gender/height-standardized measures of BP (Z-scores), given our findings that phthalate exposures in healthy children and adolescents are associated with systolic BP Z-score, and increases in BP as a sign of disease progression in CKD. The analyses will be based on the BP measurements that were done in the clinic at entry and at the follow-up visits in all patients.

Aim 2

Proteinuria was assessed as the urine PCR in a specimen in all patients enrolled in CKiD. Blood pressure was measured by validated auscultatory or oscillimetric methods in all patients and the technique was standardized in each study. We will control for BMI category, race/ethnicity, sex, and treatment for kidney disease (specifically use of angiotensin-converting enzyme inhibitor or angiotensin-receptor blockers). Analyses of persistence of proteinuria will also control for prehypertension or hypertension at study entry. For BP outcomes, we will also include BP medications as covariates. We will also examine prematurity, birth weight, and race/ethnicity as potential covariates to enhance model fit.

The assessment of proteinuria has already been performed in all

participants during the primary studies. We will examine continuous measures of proteinuria as our main outcome variables. We will also examine systolic and diastolic BP Z-scores as dependent variables. We will use mixed-effect models for repeatedly measured continuous measurements. In statistical analysis of urinary excretion of KIM-1 and NGAL, we will

again use mixed effects models with log-transformed KIM-1 and NGAL urine concentrations (because of their likely skew) as the dependent variables.

Aim 3

The two markers of oxidant stress – 8-hydroxy-deoxyguanosine (8-OHG)

and F2-isoprostane -- will be assessed using commercially available ELISA kits (Cell Biolabs Inc., San Diego CA). These assays will be performed in the Core Laboratory of the NYU CTSI with quality control of the test procedure. Both of these markers of oxidative stress are stable during prolonged storage of frozen urine specimens and, therefore, they are feasible in this proposed ancillary study.

Mixed effects models will examine trajectories of oxidative stress biomarkers in relationship with BPA and phthalate metabolite concentrations, while simultaneously examining covariates and confounders as described in the main hypothesis. The alternative hypothesis will examine oxidative stress biomarkers as dependent variables in linear regression with simultaneously measured BPA and phthalate metabolites as the main independent variables, and the confounders/covariates described above. We anticipate analyzing log-transformed 8-OHdG and F2-isoprostane concentrations, as past studies have identified skewed distribution of these variables.
