# Supplementary material for: Serially assessed bisphenol A and phthalate exposure and association with kidney function in children with chronic kidney disease in the US and Canada: A longitudinal cohort study
Source: PLoS Med. 2020 Oct 14;17(10):e1003384. doi: 10.1371/journal.pmed.1003384 (PMC7556524; doi:10.1371/journal.pmed.1003384)
Supplement: Attachment — Submitted filename: Review PLOS Medicine_R1.docx [file pmed.1003384.s013.docx]

**Requests from the editors:**

Please revise your title according to PLOS Medicine's style. Your title must be nondeclarative and not a question. It should begin with main concept if possible. "Effect of" should be used only if causality can be inferred, i.e., for an RCT. Please place the study design ("A randomized controlled trial," "A retrospective study," "A modelling study," etc.) in the subtitle (ie, after a colon).

Response: We have changed the title accordingly.

Abstract – Please add summary demographic information, including mean age and please ensure here and throughout that p values are provided for all quantifiable data and where 95% Cis are given. Please provide a brief outline of the study’s limitations as the final sentence of the ‘Methods and Findings’ section of the abstract.

Response: We have implemented these edits to the Abstract accordingly.

Data – you state that some restrictions apply, then provide a link for the data. Can you clarify what restrictions there are? Is all of the data used in your analysis in the given link?

Response: The data are owned by a third party and the authors do not have permission to share the data. This study represents the work of an ancillary study to the parent CKiD study. Access to the data may be arranged through contacting the parent study through the given link: https://repository.niddk.nih.gov/studies/ckid/.

At this stage, we ask that you include a short, non-technical Author Summary of your research to make findings accessible to a wide audience that includes both scientists and non-scientists. The Author Summary should immediately follow the Abstract in your revised manuscript. This text is subject to editorial change and should be distinct from the scientific abstract. Please see our author guidelines for more information: <https://urldefense.proofpoint.com/v2/url?u=https-3A__journals.plos.org_plosmedicine_s_revising-2Dyour-2Dmanuscript-23loc-2Dauthor-2Dsummary&d=DwIGaQ&c=j5oPpO0eBH1iio48DtsedeElZfc04rx3ExJHeIIZuCs&r=Mi3YFxiBDk6WKVx7FqA2xkMXL6Tidc8A9-U6OJpGgAg&m=ASSufQJkUEA07KFFQ3cMXAsmaVnYvQ7AIJNxPLYL9n8&s=S7RF-0vKXWem7zlGG26o7YgsdPP6MQgb80uCrAyHKOE&e=>

Response: We have added an Author Summary.

References – in the main text please use square brackets instead if rounded.

Response: The reference style has been changed.

Line 151- “several sites”, please for relevance provide some of the cities.

Response: We have added some specific sites for context.

Was written consent provided (from parents / guardians)?

Response: Informed consent was obtained from all parents or legal guardians and assent from all participants depending on their age and institutional guidelines. This information has been added to the text (lines 200-202).

Please provide a call-out to the analysis plan at the start of the Methods section (S1text)

Response: We have added this.

Please address the comments from the Academic Editor below as well as the referee's comments.

**Comments from an Academic Editor:**

The findings should be interpreted in the context of the fact that no associations were observed between the exposures and clinically relevant outcomes including eGFR (identified as the primary outcome), UPCR, SBP or DBP. Urinary NGAL and KIM-1 are recognised as markers of tubular injury but have not been shown to add additional prognostic value to traditional markers like eGFR and UPCR. In addition, the effect size was modest such that a 1 SD increase in exposure was associated with only a 9% increase in urinary NGAL or a 12% increase in urinary KIM-1.

Response: We agree and have highlighted throughout that although we found a robust pattern of increased tubular injury and oxidant stress, we did not find consistent associations between bisphenol A and phthalates and clinically relevant outcomes (i.e., eGFR, UPCR, SBP, and DBP).

A major limitation of the study is that assessment of chemical exposure was based on assays performed on random spot urine samples. As mentioned by the authors, the half life of these chemical is short (3-16 hours) so it is not clear whether random urine values accurately reflect 24 excretion.

Response: We thank the editor for this comment. Conducting epidemiologic studies with non-persistent chemical exposures is challenging. We note that previous studies have found high correlations between spot urine samples and 24-hour collections [1]. Furthermore, 24-hour collections do not provide markedly improved consistency over time [2] and are significantly more cumbersome to implement in a population-based study (and in this study, was not possible because we used banked biospecimen for exposure assessment). In order to take temporal variability into account in our analysis, we conducted a sensitivity analysis modeling cumulative exposure over time and results were largely consistent. Lastly, we hypothesize that this random variability in the exposure measure would likely lead to bias toward the null and thus make our estimates more conservative. Still, we acknowledge the limitation of using spot urine samples for exposure assessment of non-persistent chemicals and we have added this to the paragraph on limitations in the Discussion section (lines 441-447) as well as the Abstract.

**Comments from the reviewers:**
 **Reviewer #1: Statistical review**

This study is a cohort study examining association between exposure to bisphenol A and phthalates on various measures of kidney function and damage amongst children with chronic kidney disease.

I have some comments on the statistical methods and reporting, which are listed below.

1.      Abstract - just for clarity it should be clear how many separate phthalate metabolites were tested for association.

Response: We examined six exposure measures in relation to all outcomes: BPA, phthalic acid, and four phthalate groupings. This has been added to the Abstract.

2.      Abstract - if possible within space constraints, report the association between phthalic acid and eGFR over time as is done for the other associations above.

Response: We have added this to the Abstract.

3.      Were the groupings of phthalates pre-specified?

Response: The four phthalate groupings we examined were high molecular weight (HMW) phthalates, low molecular weight (LMW) phthalates, diethylhexyl phthalate (DEHP), and di-n-octyl phthalate (DOP). In the prospective analysis plan provided in the S1 text, we added a section on this that came directly from the grant. It specifically discusses examining HMW, LMW, and DEHP metabolites. In the manuscript, we added DOP as a grouping because it followed the guidance documented in the S1 text: to assess biomarkers in groups corresponding to their use in product categories. The DOP grouping is similar to DEHP; it is a sum of metabolites corresponding to the parent compound.

4.      Page 10 - what is the interpretation of the intra- and inter-assay CVs? They seem broadly within the rules of thumb of being below 10 and 15 respectively, but not all. What effect would higher CVs have on the analysis?

Response: The CV is a measure of variability that is calculated by dividing the standard deviation of a set of measurements by their mean, which is then expressed as a percent. The interpretation of the intra-assay CV is a measure of variability within sample replicates on the same analytic plate. The interpretation of the inter-assay CV is a measure of variability of replicates or controls between plates. A high CV would constitute increased variability, which would likely lead to a loss in precision of estimates and possibly a bias toward the null.

5.      Page 11 - I think for some cases it would be useful to explore how well the model fits the data. The authors do provide a sensitivity analysis to explore how cumulative effects of the exposures affect the clinical variables. When the results differed from the two approaches, perhaps the AIC could be used to show which model fitted better?

Response: We compared the AIC across the sets of models and in most cases, found that the fit was comparable. We have added this to the manuscript. For 8-OHdG and KIM-1, the conventional approach used in the primary analysis fit the data slightly better than using the cumulative average approach. This AIC comparison is shown in the table below.

|  |  | AIC | |
| --- | --- | --- | --- |
| Outcome | Exposure | Primary analysis (i.e., urinary concentrations at each time point) | Sensitivity analysis (i.e., cumulative average exposures) |
| eGFR | lnBPA | 15820 | 15819 |
|  | lnPA | 15817 | 15820 |
|  | lnLMW | 15814 | 15792 |
|  | lnHMW | 15821 | 15819 |
|  | lnDEHP | 15821 | 15820 |
|  | lnDOP | 15819 | 15820 |
| SBP-Z score | lnBPA | 5770 | 5770 |
|  | lnPA | 5770 | 5770 |
|  | lnLMW | 5769 | 5765 |
|  | lnHMW | 5770 | 5770 |
|  | lnDEHP | 5770 | 5770 |
|  | lnDOP | 5770 | 5770 |
| DBP-Z score | lnBPA | 5487 | 5487 |
|  | lnPA | 5486 | 5487 |
|  | lnLMW | 5483 | 5476 |
|  | lnHMW | 5486 | 5487 |
|  | lnDEHP | 5487 | 5487 |
|  | lnDOP | 5487 | 5487 |
| Ln-UPCR | lnBPA | 5284 | 5285 |
|  | lnPA | 5284 | 5285 |
|  | lnLMW | 5284 | 5278 |
|  | lnHMW | 5284 | 5284 |
|  | lnDEHP | 5283 | 5285 |
|  | lnDOP | 5285 | 5284 |
| Ln-8-OHdG | lnBPA | 4831 | 4836 |
|  | lnPA | 4766 | 4823 |
|  | lnLMW | 4762 | 4777 |
|  | lnHMW | 4738 | 4817 |
|  | lnDEHP | 4761 | 4801 |
|  | lnDOP | 4822 | 4873 |
| Ln-F_2_-Isoprostane | lnBPA | 4398 | 4414 |
|  | lnPA | 4399 | 4424 |
|  | lnLMW | 4388 | 4381 |
|  | lnHMW | 4403 | 4403 |
|  | lnDEHP | 4417 | 4404 |
|  | lnDOP | 4401 | 4420 |
| Ln-NGAL | lnBPA | 7964 | 7967 |
|  | lnPA | 7941 | 7957 |
|  | lnLMW | 7958 | 7961 |
|  | lnHMW | 7957 | 7963 |
|  | lnDEHP | 7960 | 7964 |
|  | lnDOP | 7959 | 7966 |
| Ln-KIM-1 | lnBPA | 6873 | 6882 |
|  | lnPA | 6867 | 6894 |
|  | lnLMW | 6841 | 6867 |
|  | lnHMW | 6867 | 6892 |
|  | lnDEHP | 6867 | 6880 |
|  | lnDOP | 6856 | 6843 |

6.      Page 11 - was there much missing data, and how was it handled? Were there many patients who developed ESKD, and how were they accounted for in the analysis?

Response: Missing data on covariates was rare (range=0%-7.3%). Below is the missingness map of covariates at each subject’s first visit. Row numbers correspond to subject ID. Following that is a table showing the percentage of missing data for each covariate.

| Table. Summary statistics of covariates at subjects’ first visits. | |
| --- | --- |
|  | % Missing |
| Total N=618 |  |
| Sex | 0 |
| Age | 0 |
| Race/ethnicity (%) | 0 |
| Glomerular/non-glomerular disease type | 0 |
| Birthweight | 5.5 |
| Prematurity | 4.2 |
| ARB use | 0 |
| ACEI use | 0 |
| Body mass index | 3.2 |
| Systolic blood pressure | 7.3 |
| Creatinine | 0 |

Analyses were performed using complete case analysis. We have stated this in the manuscript and included missing counts in the Table 1. When a patient developed ESKD or started renal replacement therapy, they were no longer included in the study follow-up. During the study period, this occurred in 80 (12.9%) subjects.

7.      Methods/results - for biomarkers such as NGAL and KIM-1 where multiple exposures were associated, did the authors consider models with more than one exposure included? I would recommend this might be useful if the exposures are correlated, in order to determine whether they were independent associations or not.

Response: There are two main reasons why we considered one exposure at a time. First, LMW, HMW, DEHP, and DOP actually represent groups of individual metabolites. There are overlaps between them. To visualize this, please see the table below.

Table. Phthalate metabolites and assigned groups

|  | LMW | HMW | DEHP | DOP |
| --- | --- | --- | --- | --- |
| MMP | X |  |  |  |
| MEP | X |  |  |  |
| MBP | X |  |  |  |
| MIBP | X |  |  |  |
| MCPP |  | X |  | X |
| MECPP |  | X | X |  |
| MEHHP |  | X | X |  |
| MEOHP |  | X | X |  |
| MBZP |  | X |  |  |
| MHXP |  | X |  |  |
| MHPP |  | X |  |  |
| MCMHP |  | X | X |  |
| MCHPP |  | X |  | X |
| MCIOP |  | X |  |  |
| MCINP |  | X |  |  |

Second, due in part to the first reason, some of these exposure variables are highly or moderately correlated. Please see the correlation matrix plot below. The lower diagonal part of the matrix shows the numerical estimates of Spearman correlation coefficients. Although the estimates were calculated as if the data were cross-sectional, similar degrees of correlations were observed at each time point. Including all exposures variables could lead to multicollinearity issues.

However, we thank the review for the suggestion of examining multiple exposures simultaneously in a model. Models with all exposure variables (excluding DEHP and DOP due to high correlation with HMW owing to the fact that they are constituents of HMW) and their pairwise interactions were fitted for the following outcomes: ln-NGAL, ln-KIM-1 and ln-8-OHdG. The models included random intercepts, AR(1) within-subject correlation structures, and controlled for time and other covariates as were included in the primary analyses. We focused on the effects of exposures without their interactions with time. Results are presented below.

Outcome: ln-NGAL

|  | M1 |  |  | M2 |  |  |
| --- | --- | --- | --- | --- | --- | --- |
|  | est | 95% CI | p | est | 95% CI | p |
| logBPA | 0.066 | -0.007, 0.139 | 0.074 | 0.061 | -0.012, 0.134 | 0.098 |
| logPA | 0.15 | 0.056, 0.244 | 0.002 | 0.171 | 0.081, 0.261 | <0.0001 |
| logLMW | 0.036 | -0.060, 0.132 | 0.459 | 0.017 | -0.073, 0.107 | 0.706 |
| logHMW | 0.048 | -0.042, 0.138 | 0.294 | 0.041 | -0.045, 0.127 | 0.355 |
| logBPA:logPA | 0.027 | -0.055, 0.109 | 0.528 |  |  |  |
| logBPA:logLMW | -0.05 | -0.127, 0.027 | 0.209 |  |  |  |
| logBPA:logHMW | -0.004 | -0.088, 0.080 | 0.928 |  |  |  |
| logPA:logLMW | -0.038 | -0.113, 0.037 | 0.323 |  |  |  |
| logPA:logHMW | -0.027 | -0.102, 0.048 | 0.475 |  |  |  |
| logLMW:logHMW | -0.001 | -0.085, 0.083 | 0.987 |  |  |  |

M1: including all pairwise interaction terms; AIC = 7882; BIC = 8050

M2: no interaction terms; AIC = 7876; BIC = 8010

Likelihood ratio test: M1 vs. M2, p-value = 0.450.

Outcome: ln-KIM1

|  | M1 |  |  | M2 |  |  |
| --- | --- | --- | --- | --- | --- | --- |
|  | est | 95% CI | p | est | 95% CI | p |
| logBPA | 0.074 | 0.017, 0.131 | 0.011 | 0.073 | 0.016, 0.130 | 0.012 |
| logPA | -0.012 | -0.085, 0.061 | 0.745 | 0.003 | -0.068, 0.074 | 0.936 |
| logLMW | 0.144 | 0.069, 0.219 | <0.0001 | 0.151 | 0.080, 0.222 | <0.0001 |
| logHMW | 0.14 | 0.069, 0.211 | <0.0001 | 0.122 | 0.055, 0.189 | <0.0001 |
| logBPA:logPA | 0.006 | -0.059, 0.071 | 0.868 |  |  |  |
| logBPA:logLMW | -0.009 | -0.070, 0.052 | 0.778 |  |  |  |
| logBPA:logHMW | 0.003 | -0.062, 0.068 | 0.918 |  |  |  |
| logPA:logLMW | 0.005 | -0.054, 0.064 | 0.869 |  |  |  |
| logPA:logHMW | -0.057 | -0.114, 0.000 | 0.054 |  |  |  |
| logLMW:logHMW | 0.023 | -0.042, 0.088 | 0.487 |  |  |  |

M1: including all pairwise interaction terms; AIC = 6754; BIC = 6921

M2: no interaction terms; AIC = 6745; BIC = 6880

Likelihood ratio test: M1 vs. M2, p-value = 0.662.

Outcome: ln-8-OHdG

|  | M1 |  |  | M2 |  |  |
| --- | --- | --- | --- | --- | --- | --- |
|  | est | 95% CI | p | est | 95% CI | p |
| logBPA | 0.076 | 0.045, 0.107 | <0.0001 | 0.075 | 0.044, 0.106 | <0.0001 |
| logPA | 0.063 | 0.022, 0.104 | 0.003 | 0.068 | 0.029, 0.107 | 0.001 |
| logLMW | 0.088 | 0.047, 0.129 | <0.0001 | 0.088 | 0.051, 0.125 | <0.0001 |
| logHMW | 0.111 | 0.072, 0.150 | <0.0001 | 0.108 | 0.071, 0.145 | <0.0001 |
| logBPA:logPA | 0.018 | -0.017, 0.053 | 0.314 |  |  |  |
| logBPA:logLMW | 0.026 | -0.007, 0.059 | 0.124 |  |  |  |
| logBPA:logHMW | -0.036 | -0.071, -0.001 | 0.052 |  |  |  |
| logPA:logLMW | -0.016 | -0.049, 0.017 | 0.335 |  |  |  |
| logPA:logHMW | -0.01 | -0.041, 0.021 | 0.519 |  |  |  |
| logLMW:logHMW | 0.033 | -0.002, 0.068 | 0.078 |  |  |  |

M1: including all pairwise interaction terms; AIC = 4565; BIC = 4735

M2: no interaction terms; AIC = 4562; BIC = 4698

Likelihood ratio test: M1 vs. M2, p-value = 0.184

Most pairwise interaction terms were not significant. There were a few borderline significant interaction terms, but the likelihood ratio tests all favoured the reduced models (M2) in terms of the goodness of fit to the data. In addition, when including all pair-wise interactions among the four exposure groups, we observed similar main effects for all exposures as estimated in the reduced models.

Finally, when comparing these mutually adjusted models without the pairwise interactions (i.e., M2) with the results of the primary analyses presented in the manuscript (i.e., single exposure models), we note that the measures of association for BPA and PA with all outcomes were consistent in direction and magnitude. However, the estimates for LMW and HMW with NGAL and 8-OHdG were slightly attenuated in the mutually adjusted models compared with the primary analysis (i.e., single exposure models). Despite this, these changes were only in magnitude and conclusions remained the same: LMW and HMW were associated with increases in NGAL, KIM-1, and 8-OHdG. We prefer to present the single exposure models as currently presented in the manuscript because they represent the marginal associations between the single chemical groups and the average outcomes over time. Given that this is the first study on this topic, we prefer to present the results in this way, focusing on the effects of each exposure category and their potential interactions with time. Analyses of chemical mixtures are important and will require more sophisticated methods. These methods are outside the scope of this manuscript.

James Wason

**Reviewer #2**

In their manuscript “Serially assessed bisphenol A and phthalate exposure is associated with increased tubular injury but not overt kidney function over time in children with chronic kidney disease” the authors Wu et al. rise a socio-economic health problem of great importance. Children from day 1 can get in contact with bisphenol A (BPA) and phthalic acid (PA). I agree with the authors, that there is growing abundance and access to BPA and PA, which leads to an nearly unavoidable risk for a high cumulative potentially toxic burden over time. The authors already saw the clinical need to investigate the deleterious effect of BPA and PA on kidney function in their previous study. Hence, their aim is to bring further light to the growing evidence for an association of BPA and PA with renal tubular impairment, especially in clinically overt pediatric CKD. On the one hand side, urinary concentrations of BPA and PA metabolites were neither associated with major renal endpoints (eGFR, proteinuria), nor with changes in blood pressure. Exception has to be made for PA, which was negatively associated with eGFR. On the other hand side, BPA and PA were associated with a rise in established biomarkers for tubular injury (NGAL, KIM-1) and for oxidative stress (8-OHdG, F_2_-isoprostane). This manuscript is well written.

Major Comments:

- A clear strength of the study is to cover a broad range of metabolites amongst a large number of subjects, bringing credible data to this first study, that longitudinally examines the effect of avoidable environmental toxicants on renal function, biomarkers of oxidative stress and, ultimately, of tubular injury. Thanks to a serial assessment of metabolites and biomarkers, the data about cumulative exposure is more robust. It is also well thought-out to calculate with respect to the short half-life of the metabolites.
- Simultaneous assessment of urinary metabolite concentrations as well as biomarkers for tubular injury and oxidative stress at least offers a speculative evidence for oxidative stress as a potential biologic pathway for environmental chemicals to harm kidney function. However, there is no definite proof.

Response: We agree and have termed these results as preliminary in lines 418-419.

- If the authors were not able to detect a clear renal signal after a 4-5 year follow-up, which is likely to be just too short, but try to highlight a subclinical renal injury by measuring renal biomarkers, the main question still remains unanswered: does the potential toxic effect of BPA and PA have a permanent impact of renal function? In other words, the authors need to discuss, if the extent of tubular injury (NGAL, KIM-1) exceeds the potential of tubular regeneration.

Response: We thank the reviewer for this question due to its clinical importance. However, it is impossible to say with certainty whether the tubular injury is irreversible or not without tissue assessment. Based on our data, it is apparent that injury is occurring and ongoing in association with BPA and phthalate exposure at current levels. It is likely that prolonged exposure would enhance the damage and increase the likelihood of clinically detectable changes such as proteinuria or impaired urinary concentrating capacity. This is discussed in lines 469-471.

- Samples were collected during a time period of 10 years with a mean follow-up of only 4-5 years. The awareness in society of potential toxic effects of BPA and PA as well as other similar chemicals has changed over time, so that there is risk for a certain degree of heterogeneity. This needs to be discussed.

Response: This can be examined by the following linear mixed-effect model: $Y_{ij}= \beta_{1}+a_{i}+\beta_{2}x_{i1}+ \beta_{3}\left( x_{ij}-x_{i1} \right)+\varepsilon_{ij}$, where $Y_{ij}$ is the ln-transformed urinary concentration of each exposure, $x_{ij}$ is the year of $j$-th observation for subject $i$, $x_{i1}$ is the year of 1^st^ observation for subject $i$, and $a_{i}$ is a random intercept. Hence, ($x_{ij}-x_{i1}$) represents change in years from the first observation to $j$th observation. $\beta_{2}$ represents the effect of year of enrollment on the average change in $Y_{ij}$. $\beta_{3}$ represents the longitudinal effect of year in the expected change in $Y_{ij}$ for a given subject. This can be seen from the following formula: $Y_{ij}-Y_{i1}=\beta_{3}\left( x_{ij}-x_{i1} \right)+\varepsilon_{ij}-\varepsilon_{i1}$.

In the table below, we report $\beta_{2}$ and $\beta_{3}$ estimates from the above model for each exposure.

| Exposure | Parameter | Estimate | 95% CI | p-value |
| --- | --- | --- | --- | --- |
| BPA | $\beta_{2}$ | -0.042 | -0.062, -0.022 | <0.001 |
|  | $\beta_{3}$ | 0.005 | -0.020, 0.030 | 0.707 |
| PA | $\beta_{2}$ | -0.009 | -0.029, 0.011 | 0.352 |
|  | $\beta_{3}$ | 0.195 | 0.171, 0.219 | <0.001 |
| LMW | $\beta_{2}$ | -0.040 | -0.064, -0.016 | 0.001 |
|  | $\beta_{3}$ | -0.006 | -0.028, 0.016 | 0.561 |
| HMW | $\beta_{2}$ | -0.052 | -0.074, -0.030 | <0.001 |
|  | $\beta_{3}$ | 0.054 | 0.030, 0.078 | <0.001 |
| DEHP | $\beta_{2}$ | -0.066 | -0.086, -0.046 | <0.001 |
|  | $\beta_{3}$ | 0.045 | 0.021, 0.069 | <0.001 |
| DOP | $\beta_{2}$ | -0.089 | -0.11, -0.069 | <0.001 |
|  | $\beta_{3}$ | -0.193 | -0.22, -0.169 | <0.001 |

There was indeed heterogeneity in exposure levels. For most of above exposures, we observed negative and significant $\beta_{2}$, which means on average, exposure levels across subjects decreased with time (i.e., people who enrolled in earlier years tended to have higher exposures compared with those who enrolled in later years). We also observed that within-subject exposure levels changed with time, with PA, HMW and DEHP in a positive direction, but DOP in a negative direction. We have added these results to the manuscript.

Further, in order to assess whether calendar year of enrollment could confound the relationship between exposures and outcomes, sensitivity analyses were conducted which further adjusted the models in the main analyses for the calendar year of enrollment. Results are shown below and were almost identical to the results of the primary analysis, shown in Tables 4 and 5 of the manuscript, which suggests that calendar year at enrollment did not affect our results. We have added this to the manuscript, lines 360-361.

Table. Associations between ln-transformed chemical exposures and ln-transformed tubular injury biomarkers from linear mixed-effects models. In addition to the covariates included in the main analyses, the models further controlled for the calendar year of study enrollment for each subject.

|  | ln-NGAL (N=2010) | | |  | ln-KIM-1 (N=2010) | | |
| --- | --- | --- | --- | --- | --- | --- | --- |
|  | β | 95% CI | p |  | β | 95% CI | p |
| BPA | 0.08 | 0.00, 0.15 | 0.03 | BPA | 0.11 | 0.06, 0.17 | <0.0001 |
| PA | 0.20 | 0.13, 0.28 | <0.0001 | PA^*^ | -0.01 | -0.08, 0.07 | 0.86 |
| LMW | 0.11 | 0.03, 0.19 | 0.01 | LMW | 0.21 | 0.15, 0.27 | <0.0001 |
| HMW | 0.12 | 0.05, 0.20 | 0.001 | HMW^*^ | 0.13 | 0.03, 0.22 | 0.01 |
| DEHP | 0.10 | 0.02, 0.17 | 0.009 | DEHP | 0.14 | 0.08, 0.20 | <0.0001 |
| DOP | 0.11 | 0.04, 0.19 | 0.005 | DOP^*^ | 0.31 | 0.21, 0.41 | <0.0001 |

Abbreviations: NGAL: Neutrophil gelatinase-associated lipocalin; KIM-1: Kidney Injury Molecule-1; BPA: bisphenol A; PA: phthalic acid; LMW: low-molecular weight phthalates; HMW: high molecular weight phthalates; DEHP: di (2-ethylhexyl) phthalate; DOP: di-n-octyl phthalate

All models controlled for age, gender, race/ethnicity, glomerular status, birth weight, low birth weight, premature, ARB, ACEI, BMI-Z score, SBP Z-score (all measured at each patient’s first visit), creatinine and shifted calendar year (reference year is 2005) of first observation for each subject.
^*^Exposure has significant interaction with time (p<0.05) and estimate for exposure at baseline is presented here; time-specific estimates were plotted in Figure 1.
β: estimate per SD change

Figure. Associations between ln-transformed chemical exposures and ln-transformed KIM1 by study visit. Estimates were derived from adjusted linear mixed-effects (LME) models and correspond to a 1-standard deviation change in each ln-transformed chemical exposure. The horizontal dashed line indicates 0. Black dots indicate that the estimate had a p-value <0.05; white dots indicate a p-value ≥0.05. Abbreviations: KIM-1: kidney injury molecule-1; bisphenol A; PA: phthalic acid; LMW: low-molecular weight phthalates; HMW: high molecular weight phthalates; DEHP: di (2-ethylhexyl) phthalate; DOP: di-n-octyl phthalate; B: baseline; Y1-Y5: year 1-year 5.

Table. Associations between ln-transformed chemical exposures and ln-transformed oxidative stress biomarkers from linear mixed-effects models. In addition to the covariates included in the main analyses, the models further controlled for the calendar year of study enrollment for each subject.

|  | 8-OHdG (N=2118) | | |
| --- | --- | --- | --- |
|  | β | 95% CI | p |
| BPA* | 0.06 | 0.01, 0.11 | 0.02 |
| PA* | 0.08 | 0.04, 0.12 | <0.0001 |
| LMW | 0.12 | 0.07, 0.17 | <0.0001 |
| HMW | 0.15 | 0.10, 0.20 | <0.0001 |
| DEHP | 0.18 | 0.13, 0.22 | <0.0001 |
| DOP | 0.19 | 0.12, 0.26 | <0.0001 |

Abbreviations: 8-OHdG: 8-hydroxy-2’-deoxyguanosine; BPA: bisphenol A; PA: phthalic acid; LMW: low-molecular weight phthalates; HMW: high molecular weight phthalates; DEHP: di (2-ethylhexyl) phthalate; DOP: di-n-octyl phthalate

All models controlled for age, gender, race/ethnicity, glomerular status, birth weight, low birth weight, premature, ARB, ACEI, BMI-Z score, SBP Z-score (all measured at each patient’s first visit), creatinine and shifted calendar year (reference year is 2005) of first observation for each subject.
* Exposure has significant interaction with time (p<0.05) and estimate for exposure at baseline is presented here; time-specific estimates were plotted in Figure 2.
β: estimate per SD change

Figure. Associations between ln-transformed chemical exposures and ln-transformed 8-OHDG by study visit. Estimates were derived from adjusted linear mixed-effects (LME) models and correspond to a 1-standard deviation change in each ln-transformed chemical exposure. The horizontal dashed line indicates 0. Black dots indicate that the estimate had a p-value <0.05; white dots indicate a p-value ≥0.05. Abbreviations: 8-OHdG: 8-hydroxy-2’-deoxyguanosine; bisphenol A; PA: phthalic acid; LMW: low-molecular weight phthalates; HMW: high molecular weight phthalates; DEHP: di (2-ethylhexyl) phthalate; DOP: di-n-octyl phthalate; B: baseline; Y1-Y5: year 1-year 5.

- Where all of the subjects in contact with BPA? Only 78% were detected BPA in their urine, the detection rate of PA was 100%. Can a fitting questionnaire rule out a technical difficulty to detect urinary BPA?

Response: Bisphenol A (BPA) is ubiquitous in the environment and a detection rate of 78% represents common exposure. Despite the greater detection of phthalic acid (PA), detection limits for all compounds were low which reflect the sensitive exposure assessment methods. The detection limit for BPA was 0.15 ng/ml and for phthalate metabolites, in the range of 0.02-0.5 ng/ml, which are considerably lower than those in samples measured at the CDC’s National Center for Environmental Health (i.e., where NHANES samples are analyzed) [3]. The exposure assessment methods utilized in this study were previously reported and are cited in this paper [4]. Although none are suspected, any inability to detect BPA would similarly affect the ability to detect phthalates, including PA.

- A few metabolites were excluded due to the lack of detection, thus not being relevant enough. What would be, in the context of this study, the best metabolite as a marker for chemical exposure according to the authors?

Response: Although exposure to BPA and phthalates is common, exposure is heterogeneous and varies by product categories and use patterns [5]. For example, low molecular weight phthalates are commonly found in personal care products [6], whereas high molecular weight phthalates are used in the manufacturing of vinyl plastics and are thus found in such sources as food packaging and intravenous tubing [7]. Due to this, it is not possible to identify a “best” metabolite of exposure.

- Expression and calculation of urinary biomarkers in form of urinary creatinine ratio is reasonable and should be at least addressed in the discussion.

Response: There is debate in the literature about how best to adjust for urinary dilution in multivariable models when using a measure of urinary creatinine output [8-12]. In short, by indexing to urinary creatinine, this assumes a specific relationship between creatinine and the outcome of interest, and may conflate an effect of the exposure and any potential effect of creatinine. Furthermore, one of the outcomes of interest, urinary protein: creatinine ratio, includes creatinine in the denominator. Therefore, if we standardized the exposure of interest by this same creatinine measure (i.e., indexed by creatinine), the same creatinine value would be used in both the exposure and outcome measures, which could lead to a spurious correlation between exposure and outcome due to urinary creatinine associations rather than the exposure itself [8]. Instead, controlling for creatinine as a covariate allows the model more flexibility [10] and allows for identification of separate effects for the exposure and creatinine [9]. We have added text to the manuscript to explain this analytic choice (lines 284-290).

- Furthermore, does a urinary NGAL concentration of a 1 year old child reflect tubular damage in the same extent as it is the case for a 16 year old?

Response: We cannot speculate on the regenerative capacity of the renal tubular epithelium depending on age. However, the difference between a 1 year old child and 16 year old is much less than comparing a child with an adult. Although the age range at baseline in our study was 1.4-17.9 years, the median age was 11 and interquartile range was 7.6-14.6, which reflects a relatively narrow window of middle childhood into adolescence.

- The parabolic curve of the association of PA + HMW with KIM-1 needs to be discussed.

Response: The apparent inverted U-shaped relationship between PA and HMW with KIM-1 is shown in Figure 1. We have clarified this in the text of the Results (line 343) and included additional text in the Discussion (lines 380-382).

- The statistical approach seems to be elaborated and I advise it to be checked by a statistician (incl. the supplements).

Minor comments

- A more compact title would be favourable

Response: We have changed the title according to journal guidelines as per the editor.

- @98: renal replacement therapy already comprises renal transplantation

Response: We have edited this sentence accordingly.

**To the Editor**

Thank you very much for the possibility to review this manuscript. As described in the comments to the authors, this is the first serious approach to examine the impact of ubiquitous chemicals like BPA and PA on renal function over time. Despite the lack of evidence for a striking impact of BPA and PA on renal function, mostly due to a short follow-up period, Wu et al. clearly underscore the association of BPA and PA with oxidative stress and, ultimately, with tubular injury. In this setting of a highly sensitive population, even a subclinical renal injury becomes of great importance. This study may not provide the ultimate proof to definitively proscribe BPA and PA from daily life, but it will certainly bring attention to this modifiable environmental risk factor. I am therefore convinced, that this study will be of great scientific use and I see it suitable for publication in PLOS Medicine.

**References**

1.	Christensen KLY, Lorber M, Koch HM, Kolossa-Gehring M, Morgan MK. Population variability of phthalate metabolites and bisphenol A concentrations in spot urine samples versus 24- or 48-h collections. Journal of Exposure Science & Environmental Epidemiology. 2012;22(6):632-40. doi: 10.1038/jes.2012.52.

2.	Lassen TH, Frederiksen H, Jensen TK, Petersen JH, Main KM, Skakkebæk NE, et al. Temporal variability in urinary excretion of bisphenol A and seven other phenols in spot, morning, and 24-h urine samples. Environmental Research. 2013;126:164-70. doi: <https://doi.org/10.1016/j.envres.2013.07.001>.

3.	Zota AR, Phillips CA, Mitro SD. Recent Fast Food Consumption and Bisphenol A and Phthalates Exposures among the U.S. Population in NHANES, 2003&#x2013;2010. 2016;124(10):1521-8. doi: doi:10.1289/ehp.1510803.

4.	Malits J, Attina TM, Karthikraj R, Kannan K, Naidu M, Furth S, et al. Renal Function and exposure to Bisphenol A and phthalates in children with Chronic Kidney Disease. Environmental Research. 2018;167:575-82. doi: <https://doi.org/10.1016/j.envres.2018.08.006>.

5.	Trasande L, Shaffer RM, Sathyanarayana S, Council On Environmental H. Food Additives and Child Health. Pediatrics. 2018;142(2):e20181410. doi: 10.1542/peds.2018-1410. PubMed PMID: 30037972.

6.	Sathyanarayana S. Phthalates and Children’s Health. Current Problems in Pediatric and Adolescent Health Care. 2008;38(2):34-49. doi: <https://doi.org/10.1016/j.cppeds.2007.11.001>.

7.	Schettler T. Human exposure to phthalates via consumer products. 2006;29(1):134-9. doi: 10.1111/j.1365-2605.2005.00567.x.

8.	Weaver VM, Kotchmar DJ, Fadrowski JJ, Silbergeld EK. Challenges for environmental epidemiology research: are biomarker concentrations altered by kidney function or urine concentration adjustment? Journal of Exposure Science and Environmental Epidemiology. 2016;26(1):1.

9.	Barr DB, Wilder LC, Caudill SP, Gonzalez AJ, Needham LL, Pirkle JL. Urinary creatinine concentrations in the US population: implications for urinary biologic monitoring measurements. Environmental health perspectives. 2004;113(2):192-200.

10.	Schisterman Enrique F, Whitcomb Brian W, Buck Louis Germaine M, Louis Thomas A. Lipid Adjustment in the Analysis of Environmental Contaminants and Human Health Risks. Environmental Health Perspectives. 2005;113(7):853-7. doi: 10.1289/ehp.7640.

11.	O’Brien KM, Upson K, Buckley JP. Lipid and creatinine adjustment to evaluate health effects of environmental exposures. Current environmental health reports. 2017;4(1):44-50.

12.	O’Brien KM, Upson K, Cook NR, Weinberg CR. Environmental chemicals in urine and blood: improving methods for creatinine and lipid adjustment. Environmental health perspectives. 2015;124(2):220-7.
